# DNA fingerprinting differentiation between β-carotene hyperproducer strains of *Dunaliella *from around the world

**DOI:** 10.1186/1746-1448-5-5

**Published:** 2009-06-30

**Authors:** Jorge Olmos, Leonel Ochoa, Jesus Paniagua-Michel, Rosalía Contreras

**Affiliations:** 1 Molecular Microbiology Laboratory, Centro de Investigación Científica y de Educación Superior de Ensenada (CICESE), Department of Marine Biotechnology, Ensenada, B.C, México; 2 Biotecnología Marina, PO Box 430222, San Diego, California, 92143-0222, USA

## Abstract

**Background:**

*Dunaliella salina *is the most important species of the genus for β-carotene production. Several investigations have demonstrated that *D. salina *produces more than 10% dry weight of pigment and that the species grows in salt saturated lagoons. High plasticity in the green stage and the almost indistinguishable differences in the red phase make identification and differentiation of species and ecotypes very difficult and time consuming.

**Results:**

In this work, we applied our intron-sizing method to compare the *18S rDNA *fingerprint between *D. salina *(CCAP 19/18), *D. salina/bardawil *(UTEX LB2538) and β-carotene hyperproducing strains of *Dunaliella *isolated from salt saturated lagoons in Baja, Mexico. All hyperproducer strains reached β-carotene levels of about 10 pg/cell. Optical microscopy did not allow to differentiate between these *Dunaliella *strains; however, *18S rDNA *fingerprinting methodology allowed us to differentiate *D. salina *from *D. salina/bardawil*.

**Conclusion:**

In Baja Mexico we found *D. salina *and *D. salina/bardawil *species by using intron-sizing-method. The National Center for Biotechnology Information (NCBI) *Dunaliella 18S rDNA *gene sequences were analyzed with our methodology and extraordinary correlation was found with experimental results.

## Background

*Dunaliella *was originally described by Teodoresco 1905. Since then, taxonomic studies among *Dunaliella *have identified several new species. However, even today differentiation among halophilic and carotenogenic *Dunaliella *species in both green and red stages is difficult and time consuming. In addition, some strains and species in culture collections are misnamed and have given rise to unnecessary strains and species names [1]. This confusion of strains and species names makes comparison of results by different authors difficult [2]. *D. salina *and *D. salina/bardawil *are the only reported β-carotene hyperproducer species of the genus (at least 10%), that grows in salt saturated lagoons [3]. However, a controversy still exists about identification of *D. salina/bardawil *as different species of *D. salina *[2]. Molecular identification provides a useful tool to distinguish between inter and intra-specific morphologically similar species [4,5] and mixed populations [6,7]. Intra-species identification of community members without cultivation, avoid some selective biases associated with pure culture methods [8]. Specie-specific oligonucleotides could be useful to identify either species from culture collections or from natural environments [9,10]. Slight phylogenetic and taxonomic differences in *Dunaliella *species can conceal profound differences in their potential for production of metabolites such as carotenoids [4]. To study these organisms one must discriminate them from their presumed non carotenogenic congeners and from among species with similar morphological features [11].

**Figure 1:**
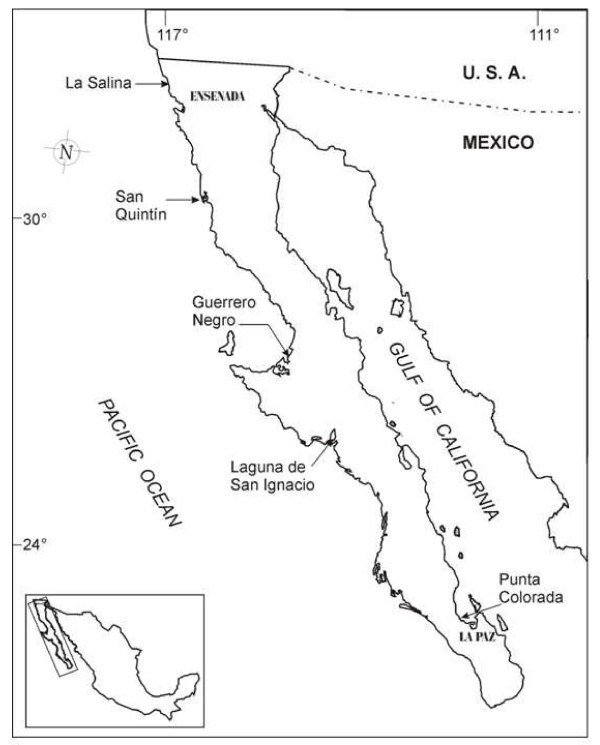
**Sampling stations of *Dunaliella *species indicated by arrows in Baja California, peninsula of México**. La Salina and San Quintín samples where used for this work.

*D. salina *strains have been found around the world (Australia, Chile, India, Israel, México, etc), however, *D. salina/bardawil *classified by Dr. Ben-Amotz has not been reported from other places than Bardawil lagoon. In this work, using intron-sizing method, we demonstrated that each hyperproducer species has an exclusive *18S rDNA *fingerprint profile. *D. salina/bardawil *species with the same *18S rDNA *fingerprint and same amount of β-carotene was found in salt saturated lagoons in Baja California Mexico (Fig. 1). Non β-carotene hyperproducer species of *Dunaliella *were easily discriminated with our methodology, even in the green stage, avoiding long cleaning, purification and growing process. The intron-sizing method provides a novel and very powerful DNA-fingerprinting technique to accomplish a specific, rapid and sensitive identification of carotenogenic *Dunaliella *species. In this work we demonstrated that auxiliary methods, such as the one proposed, could be useful obtaining well characterized and certified international culture collection.

## Results and discussion

**Figure 2:**
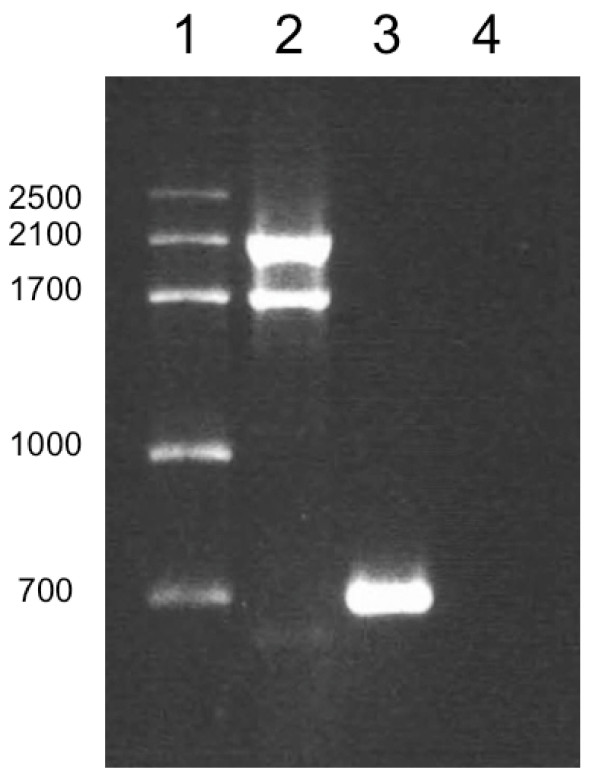
**Amplification with conserved and specific oligonucleotides**. Lane 1, molecular weight marker. Lane 2 corresponds to amplification with MA1–MA2 conserved primers, using 19/18 DNA sample. Lane 3 corresponds to amplification with DSs-MA2 specific-conserved oligonucleotides, from β-carotene hyperproducer species. Lane 4 corresponds to amplification with DSs-MA2 specific-conserved oligonucleotides, from β-carotene non hyperproducer species.

Microscopic examination of *Dunaliella salina *19/18 sample showed two different species of *Dunaliella*; one red and one green. Molecular fingerprinting determination using MA1–MA2 *18S rDNA *conserved primers from 19/18 DNA sample, gave two PCR products: one band of ~2100 bp that belongs to β-carotene hyperproducer species of *D. salina*, and a second product of ~1700 bp from *Dunaliella *species that never turns red (Fig. 2). These results agree with microscopic determination where two species of *Dunaliella *were observed (data not shown). Furthermore, using 19/18 DNA sample and DSs-MA2 specific primers we obtained a PCR product of ~700 bp from red species and no band from green species (Fig. 2). With these results we established the fingerprinting profile of 19/18 β-carotene hyperproducer strain of *D. salina *as (MA1–MA2 = ~2100 bp/DSs-MA2 = ~700 bp). Interestingly, the same fingerprinting was shown by *D. salina *M84320 isolated from Chile and reported by Wilcox and coworkers [12]. Furthermore, *D. salina *BCO2 strain isolated in Mexico, also presented the same *18S rDNA *fingerprint [5,13] as did *D. salina *strain found in India by Raja and coworkers [14]. A common characteristic from these *D. salina *strains in addition to their fingerprinting profile was their β-carotene hyperproduction capacity.

Taking into account that *D. salina *strains isolated from different continents presented the same fingerprinting profile and β-carotene levels, we can be assured that the intron-sizing method developed by our group is trustworthy, rapid and sensitive, for specifically identifying these hyperproducer strains of *D. salina*. We can conclude that *D. salina *strains with the following fingerprinting profile of MA1–MA2 = ~2100 bp and DSs-MA2 = ~700 bp, belong to the same species, are distributed worldwide and their β-carotene hyperproduction capacity is well conserved. Thus, in order to facilitate identification, we suggest a sub-classification calling these strains as "*D. salina var Teod"*.

**Figure 3:**
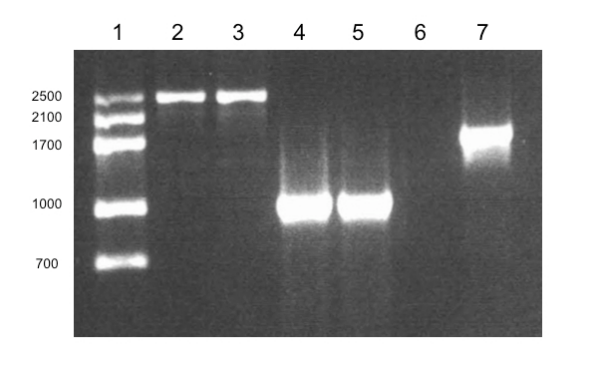
**Amplification with conserved and specific oligonucleotides**. Lane 1, molecular weight marker. Lanes 2 and 3 correspond to amplification with MA1–MA2 conserved primers, using *D. bardawil *(LB2538) and β-carotene hyperproducer species isolated from red hypersaline environments in Baja Mexico. Lanes 4 and 5, corresponds to amplification with DBs-MA2 specific-conserved oligonucleotides, from both β-carotene hyperproducers species. Lane 6 corresponds to amplification with DSs-MA2 specific-conserved oligonucleotides, from the LB2538 β-carotene hyperproducer species. Lane 7 corresponds to amplification with MA1–MA2 conserved primers, using 19/30 DNA sample.

On the other hand, both species classified as *D. salina/bardawil *purchased from UTEX (LB2538) ten years ago and *Dunaliella *strain isolated from Baja Mexico (this work), turned red and reached 10% dry weight of β-carotene. In addition, both strains amplified a PCR product of ~2500 bp using MA1–MA2 primers (Fig. 3). Specific *18S rDNA *fingerprints showed a PCR product of ~1000 bp using DBs-MA2 primers in both strains and no bands were amplified with DSs-MA2 (Fig. 3). With these results we established the fingerprinting profile of *D. salina/bardawil *as MA1–MA2 = ~2500 bp/DBs-MA2 = ~1000 bp. *D. salina/bardawil *species has not been reported from other places than Bardawil lagoon. In this sense, a controversy still exists about the authenticity of *D. salina/bardawil *as new species [2]. In addition, microscopic differentiation between *D. salina *and *D. salina/bardawil *species is difficult and time consuming. However, molecularly, *D. salina/bardawil *has an exclusive fingerprinting profile of MA1–MA2 = ~2500 bp/DBs-MA2 = ~1000 bp, different from the one presented by "*D. salina var Teod*" (MA1–MA2 = ~2100/DSs-MA2 = ~700). Certainly, more work must be done by taxonomic experts to definitively classify *D. salina/bardawil *as "*D. bardawil*" or as "*D. salina var Bardal*" or with another name. However, our methodology works well to differentiate between the two most important carotenogenic species of *Dunaliella *and to make a rapid and precise identification of them. Most importantly, this methodology is helpful in the beginning of the isolation process even in the green stage. This information is important both for commercial and scientific applications.

Recently, the National Center for Biotechnology Information (NCBI) has initiated a *D. salina *genome sequencing project (ID: 18607). In this sense, it is important to know specific identity of target species, particularly if several groups from several countries are involved in this ambitious and time consuming task. Confidence knowing specific identity of target species can avoid wasting time and money.

Additionally, demands for natural products are growing in importance and β-carotene is not an exception [1,15]. Rapid and specific identification and certification of commercial strains or species can improve production yields and help in the competitiveness by avoiding contamination with undesired species. Cross-contamination even with other hyperproducer strains still can affect the productivity due different environmental and nutritional conditions required for each strain. As an example, in May of 2008 we purchased LB2538 strain from UTEX again, but surprisingly, a fingerprinting profile of "*D. salina var Teod*" was obtained (MA1–MA2 = ~2100, DSs-MA2 = ~700) instead of the fingerprinting profile of *D. salina/bardawil *(MA1–MA2 = ~2500, DBs-MA2 = ~1000) which we were expecting (data not shown).

Moreover, contamination with non hyperproducer strains is more dangerous because these strains grow faster than hyperproducers, due to the fact that there are no metabolic efforts required for β-carotene production. This was the case for 19/18 samples where hyperproducer and non hyperproducer species of *Dunaliella *were identified by our fingerprinting methodology (Fig. 2).

*D. salina *19/30 supposedly was the strain isolated by Dr. Ben-Amotz (*D. salina/bardawil*), however PCR amplification with MA1–MA2 presented neither the ~2500 bp product associated with the original *D. salina/bardawil *species [11], nor the ~2100 bp amplification associated with *D. salina var Teod *species. Nevertheless, the 19/30 strain amplified a PCR product of ~1700 bp using MA1–MA2 conserved primers (Fig. 3) and the species itself never turns red, indicating β-carotene is not hyperproduced by this strain. In addition, Gene Bank had reported a 19/30 strain with ~1700 bp *18S rDNA *gene size (EF473749.1) and another 19/30 strain was reported with ~2100 bp *18S rDNA *gene size (DQ447648.1). The first report comes from Italy and the second comes from France. This is another good example of cross-contamination that researchers and even certified cultured collection banks can undergo, due to almost indistinguishable taxonomic characteristics between *Dunaliella *species.

Auxiliary methods like the one we are proposing could be useful for characterizing and certifying international culture collections as well. Taxonomic characterization is important to identify and differentiate species; however, differentiation between subspecies, ecotypes or similar species with taxonomy tools is a difficult and time consuming task and sometimes impossible. For this reason, the *18S rDNA *intron-sizing method proposed in this work provides a novel and powerful DNA-fingerprinting methodology to accomplish a specific, rapid and sensitive identification of carotenogenic *Dunaliella *species.

On the other hand, *D. salina *LB1644 strain obtained from UTEX did turn to red; however it produced only 1% dry weight of β-carotene. LB1644 strain amplified a ~1700 bp PCR product with MA1–MA2 conserved primers as reported by our group [5] and recently by gene bank (DQ009765.1), but did not amplify any band with DSs-MA2 or DBs-MA2. All results; *18S rDNA *gene size, fingerprinting profile and pigment production capacity, corroborated that LB1644 strain it is not *"D. salina var Teod" *n or *"D. salina/bardawil"*. In this case, more taxonomic and molecular research must be done to identify this *Dunaliella *specie. Absence of introns within *18S rDNA *gene is an inconvenience for *Dunaliella *species fingerprinting determination and identification. Fortunately, all hyperproducer species known carry I or II introns within the *18S rDNA *gene. Gómez and González [4], have been working with ITS sequences and some interesting results have been presented that could help in primer design. In addition, it seems to be the rule for hyperproducer species of *Dunaliella *to have introns within *18S rDNA*, thus "*D. salina var Teod" *has I, *D. salina/bardawil *has II and *D. parva *has II [11].

However, we did find a non carotenogenic strain in hypersaline environments that has two introns within the *18S rDNA *(data not shown). According to literature this strain could be *D. viridis*. Gene Bank sequence analysis showed a *D. viridis *strain (DQ009776) with an *18S rDNA *gene size of ~2500 bp approximately, which corresponds to the size of the strain that we found. Species-specific primer design must be done to molecularly characterize this strain and, wheatear it is possible accomplish a fingerprint profile of it.

### *Dunaliella *gene bank sequence analysis

In the past four years there has been an explosion of *18S rDNA *gene sequence submissions at Gene Bank Data Base. This sequence information is important to support the intron-sizing method developed by our group and to explain *Dunaliellas *differentiation and identification. Table 1 presents one species group of ~2500 bp which corresponds to *Dunaliella *species with two introns within the *18S rDNA *gene. Interestingly, there are only one each of *D. parva*, *D. viridis *and *D. bardawil *(*salina/bardawil*) reported. It is important to remember that *D. salina/bardawil *species from which we obtained the reported *18S rDNA *gene sequence was obtained from UTEX ten years ago. BLAST comparison showed four species with enough differences to distinguish between them and obtain an exclusive fingerprinting profile for each one (data shown only for *D. salina/bardawil*). *D. parva *β-carotene production capacity is at low levels. In addition, *D. viridis *does not produce β-carotene but can grow in hypersaline environments. Unfortunately there is no report about β-carotene production from *Dunaliella ABRII *strain (EU616729).

**Table 1:** *Dunaliella *strain sequences reported in the National Center for Biotechnology Information (NCBI) and utilized in this work.

NCBI number	year	author	strain	18S size	source	country
M62998	24-Nov-94	Lewis, L.A	Dunaliella parva LB 1983	2585 bp	Dead sea, Israel	Israel

AF150905	7-Jun-99	Olmos, J.	Dunaliella bardawil LB 2538	2584 bp	Bardawil lagoon/San Quintin	Israel/México

EU616729	29-Apr-08	Hejazi, M.A.	Dunaliella sp. ABRII G2/1	2514 bp	Gavkhouni lagoon	Irán

DQ009776	20-Dec-05	Buchheim, M.A.	Dunaliella viridis CONC002	2494 bp	Atacama Salar	Chile

						

DQ009777	20-Dec-05	Buchheim, M.A.	Dunaliella bardawil LB 2538	2088 bp	Bardawil lagoon	Israel

DQ009778	20-Dec-05	Buchheim, M.A.	Dunaliella peircei LB 2192	2065 bp	Lake Marina, Cal	USA

DQ009779	20-Dec-05	Buchheim, M.A.	Dunaliella salina LB 200	2065 bp	Dirty, salt lake	Rusia

DQ324012	1-Mar-06	Buchheim, M.A.	Dunaliella sp. SPMO 201-5	2080 bp	Salt flat, Oklahoma	USA

DQ447648	5-Apr-06	Herve, A.	Dunaliella salina CCAP 19/30	2185 bp	Bardawil lagoon	Israel

EF473739	28-Apr-07	Di Giuseppe, G.	Dunaliella salina SAG 19-3	2128 bp	Salt lake	Rusia

EF473743	28-Apr-07	Di Giuseppe, G.	Dunaliella salina CCAP 19/3	2128 bp	Dirty salt lake	Rusia

EF473745	28-Apr-07	Di Giuseppe, G.	Dunaliella salina CCAP 19/18	2151 bp	Hutt lagoon	Australia

EU583803	15-Apr-08	Hejazi, M.A.	Dunaliella sp. ABRII G2/1	2143 bp	Gavkhouni lagoon	Irán

M84320	3-Aug-94	Wilcox, L.W.	Dunaliella salina	2182 bp	Water	Chile

						

DQ324002	1-Mar-06	Buchheim, M.A.	Dunaliella sp. BSF2	2073 bp	Salt flat, Oklahoma	USA

DQ324011	1-Mar-06	Buchheim, M.A.	Dunaliella sp. SPMO 200-8	2082 bp	Salt flat, Oklahoma	USA

DQ324019	1-Mar-06	Buchheim, M.A.	Dunaliella sp. SPMO 300-5	2074 bp	Salt flat, Oklahoma	USA

DQ324020	1-Mar-06	Buchheim, M.A.	Dunaliella sp. SPMO 600-1	2074 bp	Salt flat, Oklahoma	USA

DQ324021	1-Mar-06	Buchheim, M.A.	Dunaliella sp. SPMO 601-1	2090 bp	Salt flat, Oklahoma	USA

It would be very interesting to know its β-carotene production capacities, because this strain has its own fingerprinting profile, different from the ones presented by "*D. salina var Teod*" and *D. salina/bardawil *(data not shown).

Table 1, also shows two other groups of *Dunaliella *strains with an *18S rDNA *gene size of ~2100 bp, indicating one intron within the *18S rDNA *gene. *D. salina *M84320 strain from group I, reported by Wilcox and coworkers in 1992, was used to design species-specific oligonucleotide (DSs), due its β-carotene hyperproduction capacity [5]. The *18S rDNA *fingerprinting profile from M84320 was already found in *D. salina *strains from around the world, and to make its identification easier we call these strains "*D. salina var Teod*" (data mentioned before). Furthermore, *D. salina var Teod *strains hybridized 100% with DSs primer [5'-GCAGGAGAGCTAATAGGA-3'] and hyperproduce β-carotene, data that corroborates and supports our fingerprinting results (data mentioned before).

In addition, DQ009777 strain from group I was reported as *D. bardawil*, nevertheless this data disagrees with our results for *D. salina/bardawil *where an *18S rDNA *gene size of ~2500 bp was found, instead of ~2100 bp as DQ009777 had. In addition, this strain did not amplify with the species-specific oligonucleotide (DBs) for *D. salina/bardawil*, but amplified well with DSs specific primers to *D. salina var Teod*. However, DQ009777 strain reported as *D. bardawil *by Buchheim is in agreement with the result that we obtained from *D. bardawil *(LB2538) strain obtained from UTEX in May of 2008. Nevertheless, results obtained from β-carotene hyperproducer strains isolated from hypersaline environments in Baja Mexico, demonstrate that *D. salina/bardawil *with ~2500 bp *18S rDNA *gene size exist, amplify ~1000 bp PCR product with DBs-MA2 and have an exclusive fingerprinting profile (Fig. 3). These results are in agreement with fingerprinting profile found to *D. salina/bardawil *(LB2538) obtained ten years ago from UTEX. This comparison shows that *D. salina/bardawill *from now it is not the same than 10 years ago, in the culture collection bank.

On the other hand, even when *D. salina *(DQ324002) group II strains have ~2100 bp *18S rDNA *gene size as group I had, they only hybridize 66% with DSs primer [5'-GC_t_A_c_G_t_GAGAGCTAA_g_TAGG_a_A_g_-3'], change are shown in subscript letters. Group II, therefore, belongs to a different subgroup of "*D. salina var Teod" *strains. Unfortunately we do not know if group II strains hyperproduce β-carotene. In a personal communication Dr. Buchheim mentioned that they also do not know about β-carotene production capacity of these strains. It will be important to obtain group II strains and test their β-carotene production capacity. At this moment we know that group II *Dunaliella *strains has a fingerprint of ~2100 bp with MA1–MA2 and ~750 bp with DSsII-MA2. DSsII (5-GAGAGCTAGCAGAGGGTAG-3) oligonucleotide was designed from a region 100% conserved among group II strains, using BLAST program from NCBI (data not shown).

Finally, Gene Bank BLAST analysis showed a group of *Dunaliella *strains with ~1700 bp *18S rDNA *gene size (data not shown). However, these strains were not included for group comparison because they do not have introns within the *18S rDNA *gene and do not produce β-carotene, with the exception of the LB1644 *Dunaliella *strain. It is important to remember that our fingerprint intron-sizing method depends exclusively on introns within the *18S rDNA *gene.

### Environmental strains

**Figure 4:**
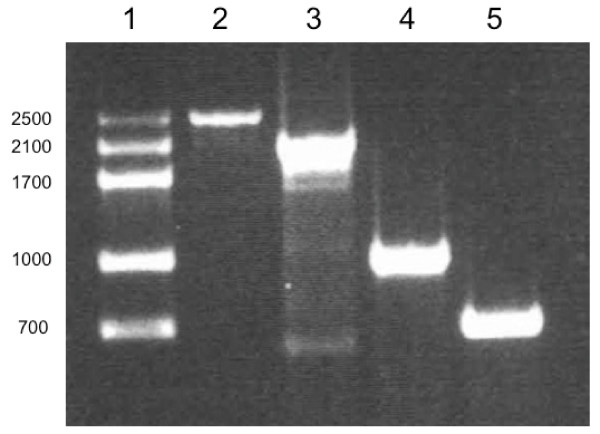
**Amplification with conserved and specific oligonucleotides**. Lane 1, molecular weight marker. Lane 2 and 3 corresponds to amplification with MA1–MA2 conserved primers, using DNA samples from San Quintín and La Salina, respectively. Lane 4 and 5, corresponds to amplification with DBs-MA2 and DSs-MA2 specific-conserved oligonucleotides, using both β-carotene hyperproducers strains.

Morphological identification demonstrated that red environmental samples contained *Dunaliella *strains (data not shown). Microscopic species differentiation was not possible due great visual similarities among *Dunaliella *species. Molecular characterization was carried out to obtain *18S rDNA *fingerprinting profile from isolated strains. Standard PCR protocol using *18S rDNA *gene conserved primers [5,11], was successfully utilized to amplify target *rDNA *region from the isolated *Dunaliella *strains. Two different sizes of DNA products were found in PCR reactions, using the same pair of conserved primers. Fragments around ~2500 bp long were found in samples coming from San Quintín B.C. In addition, fragments around ~2100 bp long were found in samples from La Salina (Fig. 4). In previous works, Wilcox and coworkers in 1992 demonstrated that microalgae strains with two introns inside *18S rDNA *have a gene size of ~2500 bp long. Additionally, the same work showed that strains with one intron within the *18S rDNA *have a gene size of ~2100 bp long. In this sense, PCR products amplified from red lagoons in Baja, Mexico, belong to *D. salina/bardawil *(~2500 bp) and "*D. salina var Teod" *(~2100 bp) respectively [5,11].

### PCR identification with *18S rDNA *specific primers

To corroborate the presence of the most biotechnologically important *Dunaliella *species in isolated samples, a standard PCR protocol was carried out with species-specific *D. salina/bardawil *(DBs-MA2) and "*D. salina var Teod*" (DSs-MA2) primers. Because introns of the *18S rDNA *gene represent the highest sequence divergence in *Dunaliella *[11,12], specific-primers were designed from them. As we expected, using DBs-MA2 and DSs-MA2 pairs of primers, two different DNA products were found in PCR reactions. Fragments around 1000 bp long were found in samples coming from San Quintín. These results corroborates *D. salina/bardawil *presence in red environmental samples coming from this place (Fig. 4) On the other hand, fragments around 700 bp long were found in samples coming from La Salina, corroborating the presence of "*D. salina var Teod*" in the sample mentioned above (Fig. 4). Results obtained with conserved and specific primers match and corroborate presences of *D. salina/bardawil *and "*D. salina var Teod*" in the environmental samples.

### Sequencing *18S rDNA *genes

The PCR product of around 2500 bp coming from the San Quintín sample using MA1–MA2 conserved primers was sequenced and aligned. BLAST results indicated that *D. salina/bardawil *reported (AF150905), matched 99% with our sequence. In addition, a ~2100 bp long PCR product amplified from the sample coming from La Salina was sequenced and aligned. BLAST results corroborated "*D. salina var Teod" *reported (M84320) matched 99% with our sequence.

### β-carotene quantification

Cell counts gave around 1–3 × 10^4 ^cells/ml in samples analyzed using a Neubauer chamber. Salt saturation was evident due to crystal precipitation. Red pigmentation was observed with high intensity, indicating great amounts of β-carotene (data not shown). Temperatures differed according to locations, being cooler in the North part of the Peninsula (20°C), than in the South (22°C). β-carotene quantification of the molecularly identified *Dunaliella *strains was developed according to conditions mentioned in materials and methods. β-carotene production reached almost same levels in all *Dunaliella *strains analyzed (~10 pg/cell).

## Conclusion

**Figure 5:**
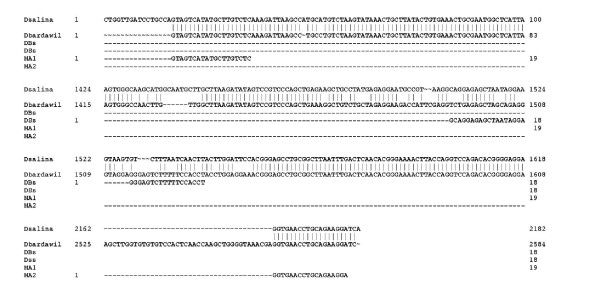
***18S rDNA *full gene sequence comparison between *D. salina *and *D. salina/bardawil***.

Using our intron-sizing method we demonstrated that "*D. salina var Teod" and D. salina/bardawil *are two different species of *Dunaliella *that could easily be differentiated by size and sequence of the *18S rDNA *gene (Fig. 5). These two species of *Dunaliella *produce nearly same amounts and profile of carotenoids, but they are difficult to distinguish phenotypically. More work in taxonomic identification must be done to classify *D. salina/bardawil *as *D. bardawil *as "*D. salina var Bardal*" or with another name.

Table 1, shows three groups of *Dunaliella *strains. The first group belongs to *Dunaliella *with two introns and approximately 2500 bp *18S rDNA *gene size. Four strains had species-specific sequence differentiation between them and as a consequence a species-specific fingerprinting profile for each one (data shown only for *D. salina/bardawil*). Table 1 also shows two groups of *Dunaliella *strains with approximately 2100 bp *18S rDNA *gene size. According to our results strains with that size contain only one intron within the *18S rDNA *gene, and are classified as *D. salina *strains. Group I *D. salina *strains (M84320 group) were called "*D. salina var Teod*", because all strains share 100% sequence conservation with DSs specific oligonucleotide, growth on hypersaline environments and hyperproduce β-carotene. In addition, these strains had been found around the world and all share the same fingerprinting profile (see results and discussion section).

A second subgroup of *D. salina *strains called group II (DQ324002 group), was identified from Gene Bank sequence analysis (data not shown). More work must be done to known their β-carotene production capacity. Additionally, even when strains of group II had the same *18S rDNA *gene size, as group I did, they only maintain 66% sequence conservation with "*D. salina var Teod" *specific oligonucleotide (DSs).

On the other hand, *D. salina/bardawil *obtained ten years ago from UTEX it is not the same strain found presently in the culture collection bank. However, a *D. salina/bardawil *strain with the original fingerprinting profile was found in San Quintín, B.C. Mexico ten years ago and also during this work.

It is important to point out that although culture collection banks may have some cross-contamination, as do collections of some researchers around the world (results and discussion), it is not our intention to point out cross contamination or incorrect affiliation of *Dunaliella *species. Nevertheless, we want to provide an auxiliary method for rapid, precise and specific identification of *Dunaliella *species.

Since our first report in 2002 on differentiation of carotenogenic *Dunaliella *species by the introns sizing method [11], we have refined, corroborated and deeply advanced the molecular differentiation of *Dunaliella *species by the fingerprinting profile method. Actually, it is the only method that has been tested on both collection bank strains as well as field samples, that allows proper identification of the physiologically plastic strains of *Dunaliella *at the level of species.

## Methods

### Type strains

*Dunaliella salina *CCAP 19/18 sample was obtained from Sams Research Services LTD, and it was originally isolated from Hypersaline Brines, Hutt Lagoon, Western Australia.

*Dunaliella salina/bardawil *was obtained from UTEX (LB2538) ten years ago. This strain was isolated from Bardawil lagoon. A strain with the same *18S rDNA *fingerprinting profile and β-carotene production capacity was isolated from Baja California, Mexico [11].

*D. salina *19/30 was obtained from sams algae collection. This strain was isolated from Bardawil lagoon. *D. salina *(LB1644) was obtained from UTEX.

Sample cells were used for morphological identification as *Dunaliella *strains and for molecular characterization as *Dunaliella *species. Cells were counted and harvested at 10,000 r.p.m. for 5 minutes. β-carotene production capacity of strains was evaluated by High Performance Liquid Chromatography (data not shown).

### Environmental strains

Two locations in Baja California peninsula of Mexico were selected for water-sample collection (Fig. 1). Environmental samples were obtained using 50 ml plastic tubes from red hypersaline waters between May and September 2006. Red microalgae strains present in those samples were morphologically identified as *Dunaliella *strains and then were molecularly characterized. Sample cells were counted and harvested at 10,000 r.p.m. for 5 minutes. β-carotene production capacity of strains was evaluated by HPLC.

### DNA purification and PCR amplifications

Isolation of chromosomal DNA from *Dunaliella *strains was carried out by the method utilized for *Escherichia coli *[16]. MA1 [5'-CG*GGATCC*GTAGTCATATGCTTGTCTC-3'] and MA2 [5-G*GAATTC*CTTCTGCAGGTTCACC-3'] conserved and DSs (*D. salina*) [5'-GCAGGAGAGCTAATAGGA-3'] and DBs (*D. salina/bardawil*) [5'-GGGAGTCTTTTTCCACCT-3'] specific oligonucleotides, were designed from *18S rDNA *genes and were previously reported by Olmos and coworkers [5,11]. In those works exons were used to design MA1 and MA2 conserved primers. In addition, DSs and DBs specific primers were designed from intron I, where most variable sequence is contained. PCR reactions were carried out in a total volume of 100 μl containing 50 ng of chromosomal DNA in TE (Tris-EDTA) buffer, pH 8 [16] and 200 ng MA1 and MA2 conserved primers. The amplification was carried out using 25 cycles in a GeneE thermocycler, with a Tm of 52°C to all reactions. One cycle consisted of 1 minute at 95°C, 1 minute at 52°C and 2 minute at 72°C. PCRs with specific primers were combined with MA2 conserved primer (DSs-MA2, DBs-MA2) and reactions were carried out under same conditions.

### Sequencing and alignment

MA1–MA2 PCR products from *Dunaliella *species were utilized to carry out a sequencing reactions, using previously reported Seq1 (5'-GGTTGATCCTGCCAGTAG-3'), Seq2 (5'-CCGGGCATTTTTGTCTGG-3'), Seq3 (5'-CTGCCAGCACCTTATGAG-3'), Seq4 (5'-GGGAGGATTGACAGATTG-3') and Seq5 (5'-GGAAGGAGAAGTCGTAAC-3') primers. DNA sequences were imported to BLAST for strains identification and to MegAlign program from DNAStar, to search for phylogenetic relationship correlations between them. *Dunaliella *Gene Bank sequences comparison analysis was carried out with BLAST program using *18S rDNA *genes with ~2500, ~2100 and ~1700 bp.

### β-carotene purification and HPLC quantification

One ml samples were centrifuged at 10,000 r.p.m. for 5 minutes, supernatant was discarded, and pellets were homogenized and disrupted in one ml of methanol. Samples were incubated on ice for one hour in the dark, centrifuged at 10,000 rpm 5 minutes, filtered and kept at -20°C until HPLC analysis.

Filtered samples and commercial standards were analyzed with an 1100 Hewlett Packard HPLC, using a 5 μm reverse phase 300SB-C18 Zorbax column 15 cm long, and 100% isocratic methanol elution with 0.5 ml/minute flux, throughout 25 minutes.

## Competing interests

The authors declare that they have no competing interests.

## Authors' contributions

JOS and JPM conceived the study, performed the analysis and wrote the manuscript. LOS sampled and obtained isolates. RCF performed some experimental work.
